# On Dropsies Connected with Suppressed Perspiration and Coagulable Urine

**Published:** 1836-01-01

**Authors:** 


### ON DROPSIES, CONNECTED WITH SUPPRESSED PERSPIRATION AND COAGULABLE URINE. By *Jonathan Osborne*, M.D. &c. Octavo, pp. 62. Sherwood and Co. Nov. 1835.

THE first part of this brochure was published in our Dublin contemporary, for January, 1834, and escaped our notice. The second part was read before the College of Physicians in January, 1835.

In the first part, Dr. O. stated that albuminous urine, when continuously secreted, ought to be considered as a symptom of disease in the kidney. Since that time, as physician to Sir P. Dun's Hospital, he has anxiously sought evidence, both adverse and favourable to the above opinion, and he has not met with a single instance of urine coagulating in a constant manner, in which *post-mortem* proofs (if examination took place) were not afforded of disease of kidney—" nor, on the other hand, an instance of disease being found in the kidney after death, in which, on taking a specimen of the urine in the bladder, it did not coagulate."

" On a review of the notes of all the fatal cases, I am also enabled to state, that the extent of the disease discovered after death has been, in every instance, in proportion to the degree of coagulation. Thus, when the urine only frothed on the application of heat, the kidneys, although gorged with blood, contained the grey, granulated structure exclusively in the outer portions of the cortical substance, and especially at the extremities of the gland; while in cases of complete coagulation, the entire cortical substance was filled, or rather superseded, by the deposition now mentioned, and the tubuli were both compressed and rendered indistinct. In some of the cases which have last occurred, I have also to mention that the emulgent veins were filled with a substance resembling the buffy coat of the blood, but of a curdy texture." 22.

Dr. Osborne has remarked that this disease, like many others, has varied in its frequency at different periods—probably in consequence of peculiar states of the atmosphere.

"The force of the circulation in this disease appears to be depressed by the action of some specific agency not as yet ascertained. The surface and extremities are uniformly cold—the latter being either livid or pallid; and, on reviewing my collection of cases, I find that in all of them the pulse was low, undulating, and ranging from sixty to ninety, except when they were complicated with inflammations; and that in those cases it was considerably less frequent than usual." 27.

And again :—

"The perspiration was either completely extinct, or confined to occasional breakings forth in the head or chest, the palms of the hand, or soles of the feet. The skin was dry and shining, harsh to the touch; and, on examining it with a lens, the usual eminences belonging to the orifices of the follicles were no longer to be found, and the orifices themselves were hardly perceptible, except when they appeared like black dots, in consequence of being filled with the residue of old secretions.

*Whenever general perspiration came on, either spontaneously, or in consequence of medicine, then the cases always terminated favourably.*" 27.

Our able author has entered into various physiological and pathological disquisitions respecting the function of perspiration, and the injurious consequences of its interruption or suppression; but these we must pass over. He must be an inattentive observer and a bad physician, who can undervalue the functions of the skin! Indeed, it is not improbable that one-fourth of the diseases of the human race originate in checked perspiration.

"On reviewing the causes of the disease in thirty-six cases, in twenty-two individuals it could be directly referred to suppressed perspiration. One of these was Thomas Leahy, a remarkably vigorous man, in his thirty-fifth year, of sober habits. It appears that he was inconvenienced by the excessive perspiration of his feet, and that at the suggestion of a friend he wore fuller's earth in his shoes in order to repress it. The effect was immediate. The perspiration ceased not only in his feet, but also in every part of his body. Diarrhœa soon came on; and, when this was subdued by appropriate remedies, universal œdema, with coagulable urine, succeeded. Although, under the treatment adopted, the œdema was removed, yet the healthy action of the skin was never restored, and I am informed that his dropsy returned. In another of those cases, the commencement of the disease was attributed to cold bathing: but the most frequent cause of it was remaining in wet clothes. As the excitement consequent on the suppressed perspiration takes place in the secreting portion of the kidney, and neither in the *tubuli*, nor in the membranes, no acute pain is perceived; and the patient is usually barely sensible of a weight in the loins, or of a thrilling sensation shooting down the thighs. Hence has arisen the obscurity which has attended the formation and establishment of this organic disease. The next frequent cause is the abuse of diuretic drinks and medicines. Of the thirty-six cases, ten occurred in confessed drinkers of ardent spirits. One of these was able to follow his trade, until the circumstances attending the fire at the Custom House afforded him an opportunity of indulging his passion for liquor. After drinking whiskey out of his hat to an extent which he was unable to define, he lay on the ground in a state of insensibility till late on the following day; and in addition to dry skin and urine frothing by heat, he exhibited a complication of ulceration of the larynx, enlargement of the liver, and violent neuralgia of one of the frontal nerves. Yet in this individual the perspiration was restored, and he was freed of the œdema, and much relieved in all other respects. The confessed drunkards in my list of cases are limited to ten; but if we could ascertain the truth respecting the mode of life of all our patients, there is no doubt that many more would have been added to this number." 33.

Diuretic medicines have also appeared to our author to be a frequent cause of the disease. Squills and the diuretic salts, though of the utmost importance in many thoracic affections, become, if long continued, the means of producing over-excitement of the kidneys, and thus a reproduction of the disease for which they were first exhibited.

"With regard to the influence of other diseases. Of the thirty-six patients, four were scrofulous; three laboured under pericarditis; and three under valvular disease of the heart. This last connexion has been placed in rather a prominent point of view by Dr. Bright. In my cases, the two diseases appeared to be combined only by both being the result of one cause, namely suppressed perspiration; and a great number of valvular diseases of the heart have occurred to me without any disease of the kidney, except the usual deficiency in secreting power; which, as a necessary consequence of impeded circulation, comes on towards the fatal termination of such diseases." 34.

Again:—

"Of the thirty-six patients, eighteen laboured under bronchitis, in different degrees of intensity; eleven had gastro-enteric inflammation, denoted by thirst, vomiting, or diarrhœa; and the two diseases were in six instances combined in the same individuals. Thus it appears that nearly two-thirds of the entire number laboured under inflammation of the mucous membranes. It is also to be observed, that in every case, before improved by treatment, the appearance of the mucus in the urine was such as belongs to irritations of the bladder and urinary passages, not forming a transparent cloud in the lower part of the vessel, as in health, but collected into dense opake flakes, and, for the most part, resting flat on the bottom of the vessel. The co-existence of those affections with the disease in question is best explained by this circumstance—that they are all the effect of the one cause, namely, *obstructed perspiration.*" 36.

The fatal cases amounted to nine, and four of these were confessed whiskey-drinkers. In almost all our author's fatal cases, life was terminated by a low form of arachnitis, as proved by dissection.

"Thus it appears, both from the causes as related in the history of the individual cases, and from the average number of the accompanying affections, that this disease is connected more especially with suppressed perspiration, than with any other known agency; but that it may also be produced by excitement of the kidneys from spirituous liquors.

Subsequent observations have convinced me that it is produced *in the most decided manner by a combination of both,* as when an habitual drinker is exposed to a long-continued application of cold." 40.

The treatment may be easily gathered from the foregoing observations. It consists chiefly in the warmth of the bed—purgatives—(avoiding diuretic aperients)—diaphoretics—warm baths—diluent drinks—bloodletting—and counter-irritation. Mercury was not found beneficial. We strongly recommend this little brochure to the attention of our brethren.

